# Stage of Change and Motivation to a Healthier Lifestyle before and after an Intensive Lifestyle Intervention

**DOI:** 10.1155/2016/6421265

**Published:** 2016-04-28

**Authors:** Buratta Livia, Reginato Elisa, Ranucci Claudia, Pippi Roberto, Aiello Cristina, Sbroma Tomaro Emilia, Perrone Chiara, Tirimagni Alberto, Russo Angelo, De Feo Pierpaolo, Mazzeschi Claudia

**Affiliations:** Healthy Lifestyle Institute, Centro Universitario Ricerca Interdipartimentale Attività Motoria (C.U.R.I.A.MO.), University of Perugia, Via G. Bambagioni 19, 06126 Perugia, Italy

## Abstract

*Objective.* Lifestyle modification programs are different but typically include both nutritional aspects and physical activity as main domains with different behavioral and/or psychological strategies designed to affect change. A fundamental role in modifying unhealthy habits is played by personal motivation for change. The present study sought to investigate, in a group of 100 overweight/obese outpatients with and/or without TMD2, treatment seeking, the effect of an intensive lifestyle program on medical measures and motivational profile for physical activity (PA) and healthy nutrition (NUTR).* Method.* Subjects participated in an intensive multidisciplinary lifestyle intervention at C.U.R.I.A.MO. Before and after the intervention, patients received a comprehensive evaluation of their clinical, anthropometric, and metabolic states and motivation to lifestyle changes.* Results.* Data showed differences before and after intervention in both medical and motivational measures. Before the intervention patients reported to be ready, open, and determined to change and gave importance to healthy habits. After the intervention patients continued to be determined but increased the actions toward the change showing a higher degree of maintenance and of acquisition of habits especially in the physical domain of the new lifestyle.* Conclusion.* Data support the notion that the motivation should be followed during all the lifestyle interventions to support the change on both domains of the lifestyle program.

## 1. Introduction

In the last few years, interventions to reduce obesity based on changing unhealthy behavior are spreading [1–3]. Lifestyle modification programs are different from each other but always include nutritional aspect and physical activity as main domains combined, sometimes, with different behavioral and/or psychological strategies aiming to afford the challenge of change. Healthy diet and regular physical activity are considered key factors in obesity and type 2 diabetes intervention for health promotion and weight reduction [4–6]. Several evidences suggest that lifestyle interventions can reach substantial weight reduction and maintenance in the long term, promoting lifestyle modifications with healthy eating and appropriate physical activity [7, 8].

However, literature on the mechanisms underlying such kind of programs is still scarce and according to many authors identifying the potential factors affecting the attendance to the programs and their outcome is crucial [9, 10]. Specifically, the literature regarding psychosocial predictors of lifestyle program is scarce and the identification of the factors helping patients to maintain the healthy behavioral changes and avoiding weight gain in the long term is considered a strategic issue [11].

Among psychological factors, a fundamental role in attending lifestyle programs and in modifying unhealthy habits is played by personal motivation for change [12–14]. Recent researches have changed the view of motivation, from a static trait to a psychological dynamic state that can fluctuate over time in relation to many interpersonal and intrapersonal factors. Motivation is thus considered an interpersonal accessible factor that can be modified during a change process [15]. Intensive interventions do not usually modify behaviors or produce deep changes but they may have a fundamental role in motivation to continue the change also in a long-term perspective [13].

In a lifestyle intervention, assessing and working on the factors affecting motivation to change seem to be fundamental in order to facilitate behavioral change [16]. Motivation can be influenced by factors as self-efficacy beliefs, moods, and social aspects which, in turn, can influence the adherence to a lifestyle program and the execution of a real and lasting behavior change [17]. According to the Transtheoretical Model (TTM) of behavior change [18], some of these factors are the motivational components influencing the transition of the subjects between the stages of the change. Within this framework motivation is considered a dynamic process involving progress through a series of five stages. The stages are temporal dimensions describing both the stage and the description of the characteristics of the subject being in that stage [19]. The five stages are as follows: (1)* Precontemplation* (P): for several reasons the subject has never thought to change her unhealthy lifestyle; (2)* Contemplation* (C): subject is thinking about a possible change because she is aware of her unhealthy habits; (3)* Determination* (D): subject has already decided to change her lifestyle and she is planning to change; (4)* Action* (A): subject is doing something to change her lifestyle; (5)* Maintenance* (M): subject in this stage commits to maintaining over time the stabilization of the change. The first two stages underline a low conviction and motivation in healthier behaviors while the last three stages indicate an involvement change. Processes of change are the covert and overt activities that people use to progress through the stages. The time of a subject's stay in a certain stage could be different. Subjects need at least three months (3–6 months) to start to change and a longer time (1 year and more) to stabilize the new acquisition. Prochaska and Velicer showed that this model allows predicting patient's participation to treatment program for health behavior [20].

If assessing motivation to change is considered a fundamental aspect in order to develop therapeutic approaches that could match the individual-specific stage of change, few papers have been devoted to examine the psychological pathways to behavior change in the domains of the lifestyle programs and literature is scarce [21, 22]. A recent study conducted with type 2 diabetes patients demonstrated that the stages of change were prerequisites to improve behaviors toward healthy diet and regular physical activity and that patients showed difficulties in recognizing the need to increase physical activity rather than eating habits. Motivation for change remained a problem for the large percentage of subjects [21]. Moreover, the high heterogeneity of response to lifestyle intervention stresses the need for the investigation of the patterns of change in physical activity and healthy diet [23].

On this basis, the aim of the present study was to explore in a group of obese,* treatment seeking* patients, participating in an intensive 3-month phase of a lifestyle program, the effects of the intervention on anthropometric, clinical, and biochemical measures and on motivational profile for physical activity (PA) and healthy nutrition (NUTR). Moreover, the aim was to investigate the existence of differences on motivational changes between PA and NUTR.

On the basis of previous literature and the characteristics of the sample, we hypothesized that at the baseline patients showed determination to change habits, in both PA and NUTR, and also that they were ready to change, giving value to beginning to achieve a correct lifestyle habits in the PA and NUTR. Considering the duration of the phase of the program investigated, we also hypothesized no changes in the motivational profile toward Action, in both PA and NUTR. No specific hypotheses have been made on differences on changes between PA and NUTR.

## 2. Measures and Method

### 2.1. Subjects Recruitment and the Research Context

The study involved 100 overweight outpatients, obese subjects with and/or without type 2 diabetes mellitus (51,1% Male and 48,5% Female), attending a lifestyle program for obesity at the C.U.R.I.A.MO. The mean age of the subjects was 51,49 (SD = 11,036) with no differences between genders (Male: mean = 52,39, SD = 12,14; Female: mean = 50,54, SD = 9,76; *F* = 0.69, *p* = 0.407). 34% of the sample had a comorbidity of type 2 diabetes. All the subjects had no orthopedic or other medical conditions that would contraindicate exercise testing or the practice of physical activity.

All the subjects participated in the intensive phase of the 3-month multidisciplinary lifestyle intervention program at the Lifestyle Institute of the University of Perugia (C.U.R.I.A.MO.) [3]. The intensive 3-month phase of intervention consists of an individualized program (in groups of five to six patients) of 26 sessions (two per week) of structured indoor exercise and nutritional counseling and eight sessions of group therapeutic education aimed at sustaining the process of lifestyle change. Before and after the intervention, patients underwent the following: (1) an initial medical examination during which they received a comprehensive evaluation of their clinical, anthropometric, and metabolic status; (2) an interview by a psychologist aimed at assessing motivation to lifestyle change; (3) an assessment by a dietician on nutritional habits; (4) a physical examination by a specialist in sports medicine on physical activity habits.

Using a quasi-experimental study design measures were collected before and after subjects' participation in the 3-month intensive phase. Data at the baseline were collected during the initial examinations; at the end of the intensive phase (±1 week) patients were asked for the same evaluations.

### 2.2. Measures

*Anthropometric, Clinical, and Biochemical Measures*. Anthropometric (height, body weight, Body Mass Index, waist circumference, and body composition), biochemical (glycaemia, HbA1c, and lipid profile), clinical (systolic and diastolic blood pressure), and strength measurements before and after intervention have been measured. Weight and body composition were performed by TANITA body composition analyzer BC-420MA. BMI was calculated as weight (kg) divided by square height (m). Waist circumference and blood pressure were measured by medical staff during clinical visits. Blood pressure was measured after 10 min at rest; the mean of 2 readings was used in statistical analysis. Biochemical analysis was performed as previously described [24].

*Motivation.* Motivation to change toward regular PA and NUTR was assessed through the use of two parallel sets of instruments belonging to the EMME-3 PA and EMME-3 NUTR based on Prochaska's TTM [12, 18]: the MAC2 R-PA and NUTR and the VMC. These measures allow assessing motivational profile in terms of stages of changes and motivational components. These measures were validated in a large study of obese and overweight subjects demonstrating good internal consistency in terms of alpha di Cronbach (EMME-3 PA: *α* range 0.68–0.87; EMME-3 NUTR *α* range 0.69–0.92).

*MAC2 R-PA and MAC2 R-NUTR.* Each of the questionnaires consists of 18 items, assessed on a Likert scale (ranging from 0 = totally false to 6 = totally true), allowing evaluating motivation to change to the five stages described above: (1)* Precontemplation* (P): for several reasons the subjects in this stage did not ever think to change their unhealthy lifestyle; (2)* Contemplation* (C): in this stage there are those subjects who think of a possible change because they are aware of their unhealthy lifestyle; (3)* Determination* (D): it is the stage where the subjects already decided to change their lifestyle and they are planning the intervention; (4)* Action* (A): in this stage there are those subjects who are doing something to change their lifestyle; (5)* Maintenance* (M): the subjects in this stage commit to maintaining over time the stabilization of the change. According to this questionnaire, the highest score indicates the prevalent stage of change.

*VMC (PA and NUTR).* The scale uses six 100-point VAS response formats to evaluate the components influencing the motivation to change: (1)* Discrepancy* (DI): it reflects dissatisfaction and concern to the present situation, need for change, and the perceived importance of change; (2)* Importance* (IM): it is the value given to correct behavior for reaching the well-being; (3)* Self-Efficacy* (SE): it reflects the perceived confidence in attaining and maintaining the predefined goals of change; (4)* Temptation* (TE): it defines the attraction by unhealthy behaviors as those that characterized the old lifestyle; (5)* Readiness to Change* (RTC): it is the degree of the problem recognition and the willingness degree to change a behavior; (6)* Stabilization of Change* (ST): it is the acquisition degree of the new lifestyle.

### 2.3. Statistical Analysis

Descriptive analysis in terms of mean and standard deviation and percentages were computed for the variables investigated for the total sample. Student's *t*-test for paired sample was used to compare anthropometric, clinical, and biochemical measures and the motivational profiles on AF and AL before and after the intensive phase of the intervention. Student's *t*-test for paired sample was also used in order to confront changes in motivational profile on AF and AL. Actual score changes, rather than scores controlling for baseline values, were used [25]. *t*-test effect sizes were also reported. According to [26] effect size *d* = 0.2 is small, those of 0.5 are medium, and those of 0.8 are large. Data were presented as mean ± SD. The data were analyzed using SPSS version 21.0.

## 3. Results

### 3.1. Anthropometric, Clinical, and Biochemical Measures

Anthropometric, clinical, and biochemical data at the baseline and after the intensive phase of the intervention are reported in Table 1. A significant decrease was found for all of the measures with a small effect size; only exception was for the LDL cholesterol and triglycerides.

### 3.2. Motivational Profile

#### 3.2.1. Physical Activity (PA): Stages of Change and Motivational Components

At the baseline subject showed the higher scores in the Determination stage (M = 84.50; SD = 17.16) indicating that patients of the sample had already decided to change toward healthier physical habits. The higher motivational component was the Importance (M = 86.06; SD = 11.29) indicating that patients gave value to the change of the unhealthy physical habits.

After 3 months the sample maintained the higher score on the Determination stage (M = 84.08; SD = 16.88) with no differences between the values before and after intervention (*t* = 0.24; *p* = 0.814). Data showed a significant decrease in Precontemplation (*t* = 2.82; *p* = 0.006; *d* = 0.32) and Contemplation (*t* = 3.61; *p* < 0.000; *d* = 0.39) stages, both with a small effect size, and an increase in Action (*t* = −6.94; *p* < 0.000; *d* = 0.91) and Maintenance (*t* = −8.96; *p* < 0.000; *d* = 1.03), in both cases with large effect size. After the intervention, the Importance remained the most influencing motivational component with the higher mean value (M = 88.93; SD = 9.85), which increases significantly with a small effect size between the values before and after intervention (*t* = 2.41; *p* = 0.018; *d* = 0.27). Increase was also observed in Self-Efficacy (*t* = −3.43; *p* < 0.001; *d* = 0.40) and Stabilization of Change (*t* = −3.75; *p* < 0.001; *d* = 0.49), also in this case both with a small effect size. Discrepancy (*t* = 6.67; *p* < 0.001; *d* = 0.83) and Temptation (*t* = 2.28; *p* = 0.025; *d* = 0.25) components decreased significantly, the first with a large effect size but the other with a small effect size. The Readiness to Change remained unchanged over time. Results are reported in Table 2.

#### 3.2.2. Healthy Nutrition (NUTR): Stage of Change and Motivational Components

At the baseline the sample showed the higher scores in the Determination stage (M = 81.25; SD = 15.50) meaning that patients had already decided to change toward healthier nutrition habits. The higher motivational component was the Importance (M = 85.80; SD = 12.75) indicating that patients gave value to the change of the unhealthy nutritional habits.

After 3-month intensive lifestyle intervention Determination remained the higher stage (M = 77.75; SD = 16.63) with no difference between the values before (M = 81.25; SD = 15.50) and after (M = 77.75; SD = 16.63) intervention. Data showed a significant before/after decrease in Precontemplation (*t* = 1.96; *p* = 0.050; *d* = 0.24) with a small effect size and in Contemplation (*t* = 5.45; *p* < 0.00; *d* = 0.67) with a moderate effect size, while data showed an increase in Action (*t* = −5.87; *p* < 0.001; *d* = 0.77), also in this case with a medium effect size, and Maintenance (*t* = −7.00; *p* < 0.001; *d* = 0.95) stage with a large effect size.

The most influencing motivational component remained the Importance (before: M = 85.80; SD = 12.75; after: M = 86.72; SD = 13.15) that did not show significant difference between the values before and after intervention (*t* = −0.83; *p* = 0.410). Only Self-Efficacy increased before and after the intervention with a small effect size (*t* = −2.05; *p* = 0.42; *d* = 0.20), whereas Discrepancy (*t* = 6.44; *p* < 0.001; *d* = 0.76) and Temptation (*t* = 4.94; *p* < 0.001; *d* = 0.50) components decreased significantly, both with a medium effect size. Readiness to Change (*t* = 1.31; *p* = 0.192) and Stabilization of Change (*t* = −0.97; *p* = 0.334) remained unchanged over time. Data are reported in Table 3.

### 3.3. Difference on Changes between PA and NUTR

The comparison between actual score changes between the two domains of physical activity and nutrition revealed no differences for the five stages of changes. The only difference, with a small effect size, was for the Stabilization of Change that was higher in PA domain (mean = 11.71; SD = 31.20) than in NUTR domain (M = 2.90; SD = 29.87) indicating that the patients had acquired a better motivational change in the physical activity domain than in nutritional habits (*t* = −2.19; *p* = 0.031; *d* = 0.29). Data are reported in Table 4.

## 4. Discussion

The aim of the present study was to investigate the impact of the intensive phase of a lifestyle program for obesity on motivational profile to change toward a healthy lifestyle in a sample of treatment seeking obese patients and to investigate possible difference in the pathways of change in the two domains (physical activity and nutritional habits) and the effects of the intervention on anthropometric, clinical, and biochemical parameters.

The intervention improved anthropometric, clinical, and biochemical measures. These results are in line with the ones of a recent meta-analysis on lifestyle programs that showed a better efficacy of diet plus exercise program on anthropometric outcomes and cardiovascular risk factors in overweight and obese participants [27]. Moreover, these results confirm previous data on the positive effects of the intensive phase of the C.U.R.I.A.MO. model on waist circumference, fat mass, and blood pressure [28].

The data of the present study showed differences before and after also on motivational profile. According to the first hypothesis, at the baseline patients showed higher score on Determination stage in both physical activity and nutritional domain. Patients of this sample showed readiness and openness to change and gave importance to behaviors toward a healthy lifestyle. The motivational components with the higher scores, in both PA and NUTR, were Importance and Readiness to Change. In Centis et al.'s findings [21], based on a sample of type 2 diabetic ambulatory patients, the majority of the sample was in the Contemplation stage, in both physical activity and nutritional domain and, as evidenced by the authors, not sufficiently aware to start the change. The fact that the patients of our study were treatment seeking in a Healthy Lifestyle Institute can explain the difference in Determination with Centis et al.'s study.

After three months subjects remained in the Determination stage in both physical activity and nutritional domain and the Determination stage did not change over the period. These results can be interpreted considering that, according to TTM, subject needs at least three months (3–6 months) to start to change and a longer time (one year and more) to stabilize the new acquisition. The 3-month duration could not be sufficient to establish the stage of Actions to a new behavioral model. Interestingly, after the intervention the two stages of Precontemplation and Contemplation decreased significantly, while the two stages of Action and Maintenance increased significantly, indicating that the intensive phase had the role to start the process of change toward a healthier lifestyle.

After the intervention the motivational components that influenced more the transition between the stages of the change, in both physical activity and nutrition, were the Importance, which increased significantly, and the Readiness to Change, which did not change over time. Among motivational components Self-Efficacy increased significantly showing that patients of this sample gained confidence and trust in their capacity to achieve the actions for the change. Self-Efficacy is a psychological component that is crucial for changing habits [14]. The probability of maintaining higher levels of physical activity and healthier dietary behavior improves when people are more self-confident in the possibility to change their behavior [29, 30].

Patients of the sample showed an increase before and after in Self-Efficacy both in physical activity domain and in nutritional area, showing that the intervention had affected the internal idea to afford effectively the challenge of changing lifestyle. Another psychological component considered crucial for changing habits is Discrepancy. After the intervention, it decreased with a large effect size. This shows that all negative feelings as pain, discomfort, and concern toward the acquisition of the new behaviors changed in positive feelings and aspiration to reach the goals previously decided, the acquisition of healthy habits [13, 15].

Regarding the existence of differences of the intervention on motivational changes between physical activity and nutrition, data showed difference only in Stabilization of Change that resulted higher on the physical activity domain. Patients of the sample showed that intervention had reinforced more the physical activity domain with a higher gain in this area of the lifestyle intervention. It is very likely that the pleasant feelings experienced by the patients during group exercise sessions reinforced the Stabilization of Change. At the end of the intensive lifestyle phase, patients with obesity attending the program showed a higher degree of acquisition in the physical domain of the new lifestyle.

Data of the present study support the idea that motivation should be evaluated and followed before and during lifestyle interventions in order to support the change, strengthening motivation of the two domains of the lifestyle program [31, 32]. The assessment of the motivation at the end of the three-month intensive phase should be considered important for the long-term sustainment of change after this initial phase.

## Figures and Tables

**Table 1:** Descriptive analysis of the obese patients at baseline and after the intervention. Data are presented as mean ± SD. Statistical significance was considered at *p* < 0.05.

Characteristics	T0	T1	*t*	*p*	Cohen's *d*
Anthropometric data					
Body Mass Index (BMI)	33.24 ± 4.64	32.21 ± 4.26	8.28	0.000	0.23
Weight (kg)	94.96 ± 17.01	91.97 ± 16.27	8.89	0.000	0.17
Fat body mass (kg)	35.07 ± 11.46	32.64 ± 10.78	6.26	0.000	0.22
Lean body mass (kg)	56.76 ± 11.93	56.21 ± 11.74	2.72	0.008	0.05
Waist circumference (cm)	109.89 ± 11.50	105.90 ± 10.56	7.19	0.000	0.36
Clinical and biochemical data					
Systolic blood pressure	132.25 ± 21.20	123.70 ± 13.84	3.93	0.000	0.48
Diastolic blood pressure	82.11 ± 8.68	73.97 ± 9.55	8.39	0.000	0.89
Glycaemia	111.17 ± 31.87	100.81 ± 24.44	6.08	0.000	0.37
HBA1c	6.24 ± 1.14	5.90 ± 0.68	3.90	0.000	0.36
Total cholesterol	200.18 ± 39.26	194.24 ± 38.87	2.21	0.029	0.15
HDL cholesterol	48.99 ± 11.81	45.48 ± 10.17	4.20	0.000	0.32
LDL cholesterol	124.37 ± 35.47	121.91 ± 32.85	0.86	0.392	n.s.
Triglycerides	143.06 ± 69.68	141.38 ± 80.31	0.27	0.788	n.s.

Statistical significance was considered at *p* < 0.05.

**Table 2:** Motivation to change to physical activity (PA) at baseline and after the intervention. Data are presented as mean ± SD.

	T0	T1	*t*	* p*	Cohen's *d*
Stages of the change					
Precontemplation	22.17 ± 21.75	16.00 ± 17.03	2.82	0.006	0.32
Contemplation	61.42 ± 23.38	52.50 ± 22.46	3.61	0.000	0.39
Determination	84.50 ± 17.16	84.08 ± 16.88	0.24	0.814	n.s.
Action	55.66 ± 31.51	80.08 ± 20.85	−6.94	0.000	0.91
Maintenance	42.75 ± 28.79	68.00 ± 18.98	−8.96	0.000	1.03
Motivational components					
Discrepancy	60.94 ± 23.36	41.65 ± 22.86	6.67	0.000	0.83
Importance	86.06 ± 11.29	88.93 ± 9.85	−2.41	0.018	0.27
Self-Efficacy	74.47 ± 14.40	79.93 ± 12.72	−3.43	0.001	0.40
Temptation	41.18 ± 21.34	35.58 ± 23.98	2.28	0.025	0.25
Readiness to Change	79.06 ± 19.36	81.88 ± 13.37	−1.24	0.215	n.s.
Stabilization of Change	55.85 ± 28.12	67.56 ± 18.30	−3.75	0.000	0.49

Statistical significance was considered at *p* < 0.05.

**Table 3:** Motivation to change to health nutrition (NUTR). Data are presented as mean ± SD.

	T0	T1	*t*	*p*	Cohen's *d*
Stages of the change					
Precontemplation	37.00 ± 20.12	31.92 ± 22.36	1.96	0.050	0.24
Contemplation	71.50 ± 21.55	56.83 ± 22.46	5.45	0.000	0.67
Determination	81.25 ± 15.50	77.75 ± 16.63	1.90	0.060	n.s.
Action	57.58 ± 26.33	74.75 ± 17.06	−5.87	0.000	0.77
Maintenance	43.08 ± 23.87	63.92 ± 19.79	−7.00	0.000	0.95
Motivational components					
Discrepancy	57.90 ± 22.04	41.27 ± 21.57	6.44	0.000	0.76
Importance	85.80 ± 12.75	86.72 ± 13.15	−0.83	0.410	n.s.
Self-Efficacy	71.71 ± 18.45	75.19 ± 15.47	−2.05	0.042	0.20
Temptation	64.43 ± 23.47	52.88 ± 22.55	4.94	0.000	0.50
Readiness to Change	78.27 ± 18.58	75.55 ± 18.40	1.31	0.192	n.s.
Stabilization of Change	61.71 ± 26.38	64.61 ± 20.41	−0.97	0.334	n.s.

Statistical significance was considered at *p* < 0.05.

**Table 4:** Paired Student's *t*-test Δ score of stage of change and motivational habitual physical activity and healthy nutrition. Data are presented as mean ± SD. Actual score changes are reported.

	Δ score PA	Δ score NUTR	*t*	*p*	Cohen's *d*
Stages of the change					
Precontemplation	6.17 ± 21.86	5.08 ± 25.89	0.36	0.722	n.s.
Contemplation	8.92 ± 24.71	14.67 ± 26.91	−1.64	0.105	n.s.
Determination	0.42 ± 17.66	3.50 ± 18.39	−1.19	0.237	n.s.
Action	−24.42 ± 35.20	−17.17 ± 29.25	−1.82	0.071	n.s.
Maintenance	−25.25 ± 28.16	−20.83 ± 29.74	−1.22	0.223	n.s.
Motivational components					n.s.
Discrepancy	19.28 ± 28.89	16.63 ± 25.82	0.68	0.500	n.s.
Importance	−2.87 ± 11.92	−0.93 ± 11.24	−1.22	0.225	n.s.
Self-Efficacy	−5.46 ± 24.56	−3.48 ± 16.94	−0.88	0.379	n.s.
Temptation	5.59 ± 24.56	11.54 ± 23.35	−1.80	0.075	n.s.
Readiness to Change	−2.82 ± 22.60	2.82 ± 20.72	−1.87	0.064	n.s.
Stabilization of Change	−11.71 ± 31.20	−2.90 ± 29.87	−2.19	0.031	0.29

Statistical significance was considered at *p* < 0.05.
